# BK viraemia as a cause of anaemia in after ABO-incompatible liver transplant: a case report

**DOI:** 10.1186/s12879-023-08601-5

**Published:** 2023-09-18

**Authors:** Lucy McMullen, Liane Khoo, Lyndal Anderson, Simone Strasser, David M Gracey

**Affiliations:** 1 Renal Unit, Royal Prince Alfred Hospital, Camperdown, NSW Australia; 2 University of Sydney School of Medicine, Camperdown, Australia; 3 Institute of Haematology, Sydney Local Health District, Royal Prince Alfred Hospital, Camperdown, NSW Australia; 4 Department of Tissue Pathology and Diagnostic Oncology, Royal Prince Alfred Hospital, Sydney, Australia; 5 A W Morrow Gastroenterology and Liver Centre, Royal Prince Alfred Hospital, Camperdown, NSW Australia

**Keywords:** Anaemia, BK viraemia, Liver transplant

## Abstract

**Background:**

While anaemia following liver transplant is common, anaemia in the context of BK viraemia is not a commonly recognised phenomenon.

**Case presentation:**

We present the case of 59-year old gentleman with severe anaemia in the context of BK viraemia and nephropathy following ABO incompatible liver transplant. Severity of anaemia appeared to correlate with high titres of BK virus in the serum. Bone marrow biopsy revealed hypocellular marrow with normal cytogenetics. Anaemia improved with treatment with cidofovir, intravenous immunoglobulin, reduction in immunosuppression and erythropoietin stimulating agent.

**Conclusion:**

To our knowledge, this is the first case of anaemia post liver transplant contributed to by BK viraemia.

## Background

Anaemia post liver transplant is common, with rates ranging from 4.3 to 58% depending on criteria and time frame used to define anaemia [1, 2]. Common causes of anaemia post liver transplant include viral infections (such as Parvovirus B19 infection and cytomegalovirus); impaired renal function, and impaired bone marrow function due to toxicity of drugs used in the post-transplant period. Immunosuppressant drugs commonly used in the post-transplant period known to suppress bone marrow activity include mycophenolate, azathioprine, and mammalian target of rapamycin inhibitors [2]. Valganciclovir and cotrimoxazole used in infection prevention or treatment also contribute to anaemia [2]. Dose reduction or cessation of immunosuppressants due to toxicity increases the risk of acute rejection, and withdrawal of prophylactic medications increases the risk of infections. Rarer causes of anaemia after liver transplant include aplastic anaemia, graft versus host disease and lymphoproliferative disorders [2]. The aetiology of anaemia is likely to be multifactorial for many patients. In one study, the cause of anaemia was unidentifiable despite extensive investigation in 47% of patients [3].

The human polyomavirus is known to be associated with polyomavirus-associated nephropathy and ureteric stenosis, which occurs most commonly in renal transplant patients. BK virus is known to replicate in epithelial urinary cells, and has a tropism to vascular endothelial cells [4, 5]. BV virus also replicates in peripheral blood mononuclear cells [4], and there is increasing recognition of haematologic effects of the virus. A case of BK virus related haemophagocytic syndrome has occurred in a kidney transplant patient [6]. BK virus replication has been observed in the bone marrow and blood of kidney-transplant patients [7, 8].

Here, we present a case of BK viraemia-associated anaemia post liver transplant.

### Case presentation

A 59-year old Vietnamese-born man received an ABO-incompatible orthoptic liver transplant for liver failure due to hepatitis B reactivation. Complications following transplant included acute kidney injury due to biopsy confirmed severe BK nephropathy of the native kidney with considerable interstitial inflammation. He had concurrent biopsy-proven cytomegalovirus colitis, and T-cell mediated rejection. Throughout the post-transplant course, relapsing normocytic normochromic anaemia occurred.

**Fig. 1:**
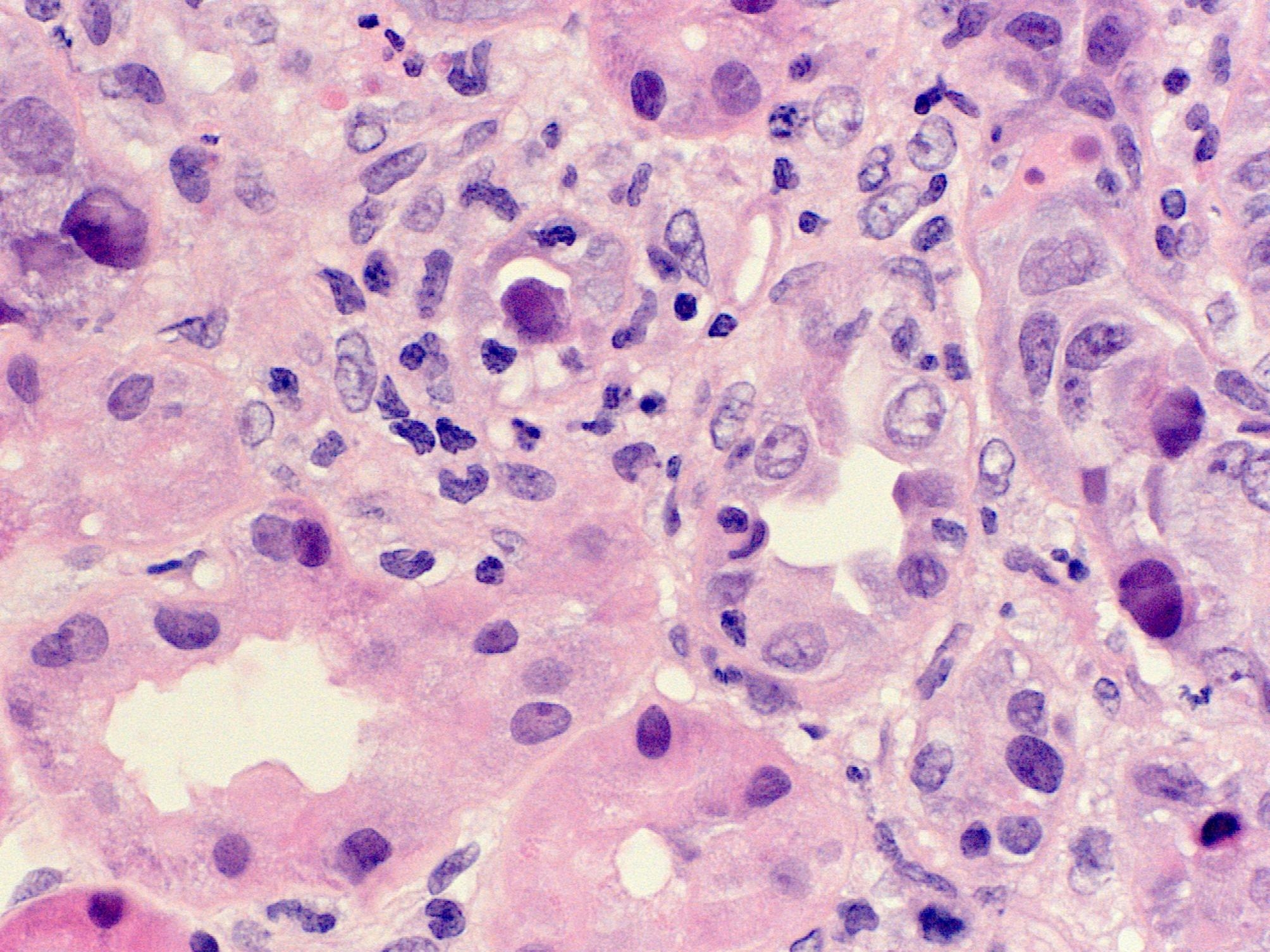
Light microscopy of native renal biopsy (haemotoxylin and eosin stain, magnification x400) displaying enlarged tubular nuclei with viral inclusions

**Fig. 2:**
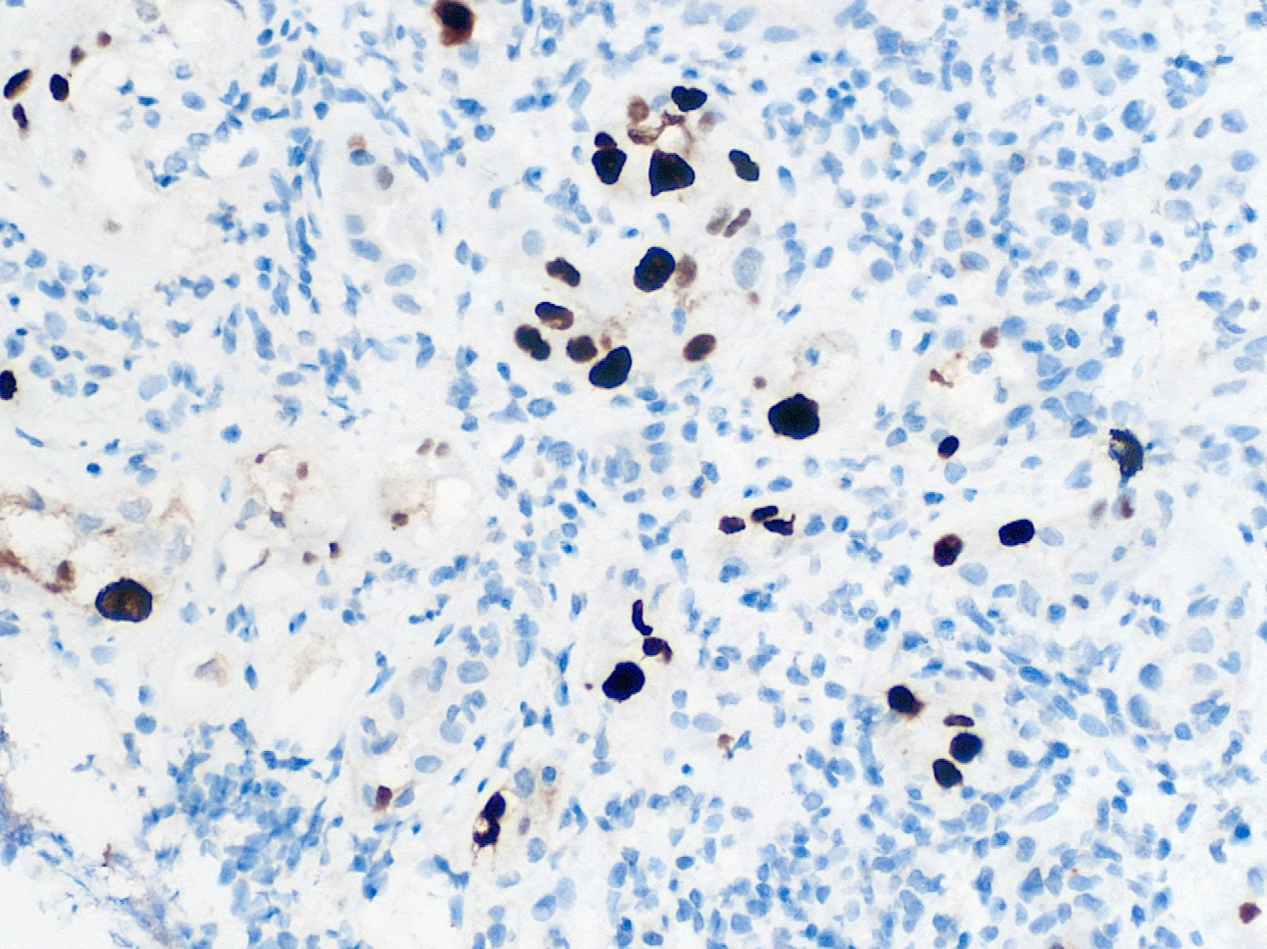
Abnormal tubular nuclei stained positive for SV-40 (polyomavirus) on immunoperoxidase (magnification x200)

Diagnosis of biopsy-proven BK nephropathy (Figs. 1 and 2) occurred twenty-two months post-transplant with 754 976 copies/mL, which was previously reported in Transplantation [9]. At diagnosis, creatinine was 297 mg/dL with an eGFR of 19 mL/min/1.73m^2^; and the haemoglobin was 73 g/L. After treatment of BK virus with reduction in immunosuppression and a 12-week course of cidofovir at a dose of 0.25 mg/kg, and treatment of CMV colitis with 7 days of intravenous ganciclovir followed by oral valganciclovir at a renally adjusted dose, haemoglobin improved to a baseline of 100 g/L. His renal function also improved to a creatinine of 220 mg/dL and eGFR of 27 mL/min/1.73m^2^, which has been sustained since.

Two months later, five doses of intravenous methylprednisolone were administered for biopsy confirmed mild acute T-cell mediated rejection, and tacrolimus was increased. Following this increase in immunosuppression, BK viraemia rose to 26 342 copies/mL and anaemia recurred with a haemoglobin of 60 g/L. His anaemia was treated with transfusion of one unit of packed red blood cells, and haemoglobin increment was sustained with a second course of cidofovir and commencement of monthly methoxy polyethylene glycol-epoetin beta. Mycophenolate was then changed to everolimus.

Our patient had a stable course for ten months, during which tacrolimus was ceased and mycophenolate was reintroduced to reduce the risk of calcineurin inhibitor nephrotoxicity.

**Fig. 3:**
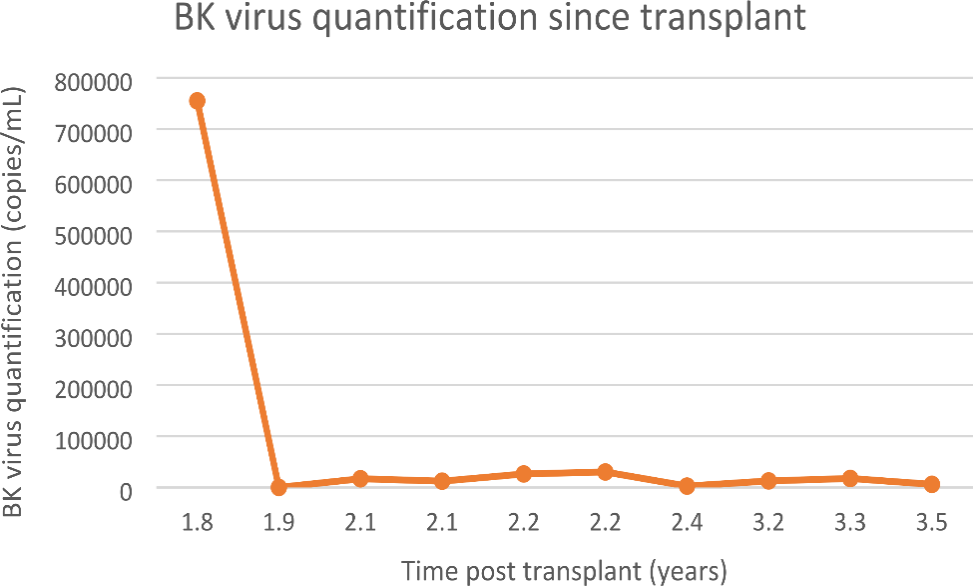
BK Virus quantification since transplant

**Fig. 4:**
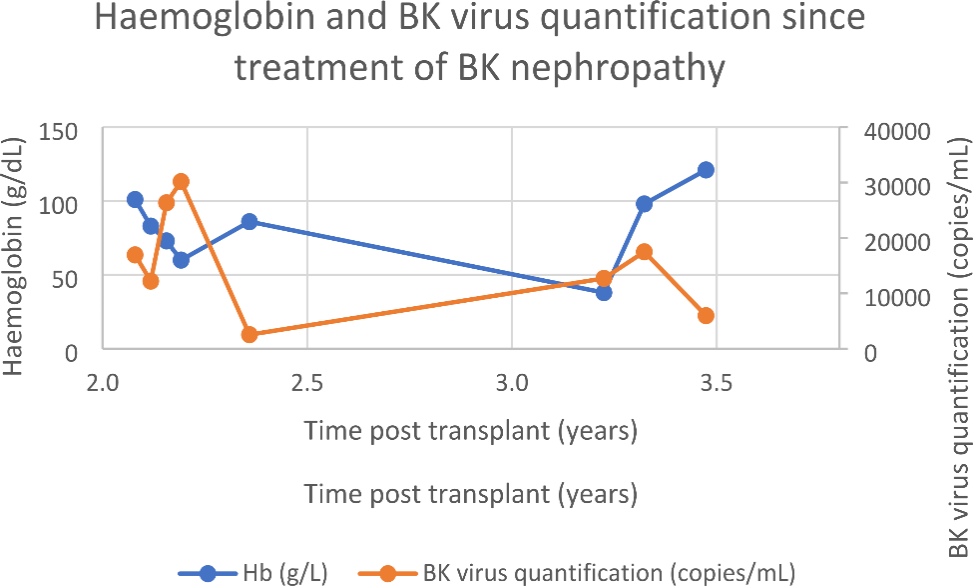
Relationship of haemoglobin and BK virus quantification since transplant

Three years post-transplant, our patient was admitted after routine bloods revealed a haemoglobin of 38 g/L. There was a four week history of fatigue and shortness of breath, though no symptoms of blood loss. Examination did not show hepatosplenomegaly. The immunosuppressive regime was everolimus 2 mg bd and mycophenolate 1 g bd, and he remained on monthly erthropoietin stimulating agent. Investigations demonstrated no abnormality of other cell lines, and renal and liver function were at the patient’s baseline. BK viraemia had recurred at 12 700 copies/mL. There were no signs of haemolysis, myeloma screen was negative, CD59 + expression was normal, parvovirus IgM was negative and there was no CMV viraemia. Bone marrow biopsy showed moderately hypocellular bone marrow with moderately reduced erythropoiesis and mild dysplastic features, reduced granulopoiesis, and dysplastic megakaryocytes thought to be due secondary to either drugs, infection or inflammation. Retrospective staining for BK virus on the bone marrow aspirate was negative. The cytogenetics on the bone marrow were normal. The anaemia was treated with blood transfusion, four doses of intravenous immunoglobulin, and a third course of cidofovir, and continuation of erythropoietin stimulating agent. The haemoglobin incremented to 118 g/L and mycophenolate dose was reduced. On completion of this course of cidofovir, the haemoglobin was 121 g/L and BK titre reduced to 5980 copies/mL. BK virus quantification from transplant to present is seen in Fig. 3, and the relationship of BK viraemia following initial diagnosis to degree of anaemia is shown in Fig. 4.

## Discussion and conclusions

Classically, complications of polyomavirus include ureteric stenosis and polyomavirus associated nephropathy in renal transplant patients; and polyomavirus associated haemorrhagic cystitis in recipients of an allogeneic haemopoietic stem cell transplant [4].

BK virus infection of the native kidney after liver transplant is rare, though possibly underrecognised, with only three cases previously reported to the best of our knowledge [9–11]. Recognition of haematological manifestations of polyomavirus are increasing. In humans, BK virus replication is known to occur in both blood and bone marrow [7, 8].

Our patient, who had not previously had acute episodes of anaemia, had repeated acute episodes of anaemia with contemporaneous high BK virus titres. In this case, relapse in degree of BK viraemia between periods of treatment with intravenous immunoglobulin and cidofovir appeared to proceed presentations with severe symptomatic anaemia. Anaemia also appeared to improve on treatment of BK viraemia with reduction in immunosuppression, and administration of intravenous immunoglobulin and cidofovir. While our patient’s anaemia is also contributed to by chronic renal impairment, there appears to be a temporal relationship of anaemia following periods of high BK viral load.

As far as we are aware, this is the only documented case of anaemia post liver transplant with BK viraemia as a contributor.

Anaemia associated with BK viraemia post renal transplant has previously been documented in a study of 72 renal transplant recipients (Pambrun et al.), and two case reports [7, 12].

In a study by Pambrun et al., renal transplant recipients who presented for investigation of cytopenia we screened prospectively for BK virus in the blood and bone marrow. Bone marrow biopsy was performed when haemoglobin had decreased to < 11 g/dL, and/or neutrophil count was < 1000/mm3 and/or platelet count was < 120,000/mm3. BK viraemia was detected in the blood of 9 of 72 renal transplant recipients. Only 5 of these patients had BK virus replication in the bone marrow. Interestingly, BK virus replication was detected in the bone marrow of an additional three patients who had no detectable BK virus replication in the blood, therefore 8 of 72 patients (11.1%) exhibited bone marrow replication of the virus. Most of the patients who had BK virus detected in the marrow presented for investigation of neutropenia, and hypocellularity of the myelopoietic line and blockade of granulocyte maturation were the most common lesions observed in bone-marrow analyses [8]. Similar features were present in our patient’s bone marrow aspirate, however retrospective testing for BK virus replication in our patient’s marrow was negative.

One case describes 17 year old renal transplant recipient who was treated for BK virus nephropathy presented with pancytopenia, with BK virus detected in blood and bone marrow at high viral loads. Graft nephrectomy and immunosuppression improved the cytopenias [7].

Another case describes anemia after renal transplant in the context of concurrent parvovirus B19 and BK viraemia [12]. Sharma et al. proposed that the anaemia was primarily associated with parvovirus rather than BK virus, as the peak plasma BK level in their case was 6234 copies/mL, which is unlikely to cause any renal dysfunction or urological issue. This compares with a peak of 754, 976 copies/mL in our case. Further, parvovirus B19 infection has been more commonly recognised as a cause of anaemia post-transplant than polyomavirus.

The exact mechanism by which BK viraemia causes anaemia remains unclear. In mice with severe combined immunodeficiency, acquiring BK virus resulted in an acute haematological disorder causing haemorrhage, anaemia, thrombocytopenia and splenomegaly. Authors propose an indirect method of myeloproliferation such as secretion of growth factors or cytokines by virus-infected cells [13]. Pambrun et al. speculate that BK virus infects granulocyte progenitors by trafficking through the cytoplasm toward the cell nucleus, where the uncoated viral genome utilises cellular machinery to replicate.

In summary, this is the first case of post-liver transplant anaemia, which is significantly contributed to by BK viraemia. The association of anaemia and BK virus replication in the marrow raises the need to consider bone marrow biopsy with staining for BK virus in resistant cases of anaemia post solid organ transplant, even in the absence of BK virus replication in the blood.

## Data Availability

The patient specific data analysed in this case report is not publicly available for patient privacy reasons, however can be made available from the corresponding author on reasonable request.

## References

[1] Berger T, Reisler I, Shochat T, Raanani P, Nesher E, Mor E (2020). Post-Liver Transplantation Anemia and its correlation with mortality and graft failure. Dig Dis Sci.

[2] Maheshwari A, Mishra R, Thuluvath PJ (2004). Post-liver-transplant anemia: etiology and management. Liver Transpl.

[3] Kimka Ndimbie O, Frezza E, Jordan JA, Koch W, van Thiel DH (1996). Parvovirus B19 in anemic liver transplant recipients. Clin Diagn Lab Immunol.

[4] Hirsch H, Steiger J, Polyomavirus BK (2003). Lancet Infect Dis.

[5] Hirsch H, Randhawa P, Practice (2013). AIDCo. BK polyomavirus in solid organ transplantation. Am J Transpl.

[6] Esposito L, Hirsch H, Basse G, Fillola G, Kamar N, Rostaing L (2007). BK virus-related hemophagocytic syndrome in a renal transplant patient. Transplantation.

[7] Gardeniers S, Mekahli D, Levtchenko E, Lerut E, Renard M, van Damme-Lombaerts R (2010). Bone marrow aplasia and graft loss in a pediatric renal transplant patient with polyomavirus nephropathy. Pediatr Nephrol.

[8] Pambrun E, Mengelle C, Fillola G, Laharrague P, Esposito L, Cardeau-Desangles I (2014). An Association between BK Virus Replication in Bone Marrow and Cytopenia in kidney-transplant recipients. J Transpl.

[9] Lai C, Bleasel J, McGrath J, Majumdar A, Kirwan P, Anderson L (2020). BK Nephropathy as a cause of renal dysfunction in an ABO-incompatible liver transplant patient. Transplantation.

[10] Zeng Y, Magil A, Hussaini T, Yeung CK, Erb SR, Marquez-Alazagara V (2015). First confirmed case of native Polyomavirus BK nephropathy in a liver transplant recipient seven years post-transplant. Ann Hepatol.

[11] Sekulic M, Sloan R, Guo S, Anderson MD, Markowitz GS (2021). BK Virus Nephropathy in the native kidney of a liver transplant recipient. Kidney Int Rep.

[12] Sharma N, Bajwa R (2020). Parvovirus infection-related Anemia after kidney transplantation. Case Rep Transplant.

[13] Szomolanyi-Tsuda ED, Joris PL, Schultz I, Woda LD, Welsh BA, Acute RM (1994). Lethal, Natural Killer Cell-Resistant Myeloproliferative Disease Induced by Polyomavirus in severe combined immunodeficient mice. Am J Pathol.

